# Volatile Organic Compounds Derived from 2-Keto-Acid Decarboxylase in *Microcystis aeruginosa*

**DOI:** 10.1264/jsme2.ME12099

**Published:** 2012-10-05

**Authors:** Masateru Hasegawa, Akito Nishizawa, Kiyomi Tsuji, Shigenobu Kimura, Ken-ichi Harada

**Affiliations:** 1 Graduate School of Environmental and Human Science and Faculty of Pharmacy, Meijo University, Tempaku, Nagoya, Aichi, 468–8503 Japan; 2 VBL, Graduate School of Science and Engineering, Ibaraki University, Hitachi, Ibaraki, 316–8511, Japan; 3 Kanagawa Prefectural Institute of Public Health, Shimomachiya, Chigasaki, Kanagawa, 253–0087, Japan; 4 Department of Biomolecular Functional Engineering, Ibaraki University, Hitachi, Ibaraki, 316–8511, Japan

**Keywords:** cyanobacteria, volatile organic compounds, 3-methyl-1-butanol, 2-keto-acid decarboxylase, *Microcystis*

## Abstract

Volatile organic compounds (VOCs), 2-methyl-1-butanol, 3-methyl-1-butanol and 2-phenylethanol, were detected together with β-cyclocitral from the cyanobacterium *Microcystis aeruginosa* NIES-843. These alcohols were optimally produced after 35 d of culture, during which nitrate nitrogen in the cultured broth became exhausted. Additionally, these alcohols were definitely produced using the 2-keto-acid decarboxylase (MaKDC) in *Microcystis* strains. These results suggested that these VOCs from *Microcystis* are significant for their lifecycle, because these compounds are not produced by any other genus of cyanobacteria. This is the first report of 2-keto-acid decarboxylase producing 3-methyl-1-butanol and 2-phenylethanol by an oxygenic photosynthetic microorganism.

Blooms and scum of the cyanobacterium (blue-green alga) *Microcystis* can be a major hazard in recreational lakes, drinking water reservoirs, slow-flowing rivers and wetland areas (5, 17, 27, 28). *Microcystis* can produce a musty odor, and hepatotoxic microcystin poses serious health risks for animals and humans (4, 6); therefore, it is highly desirable to regulate the occurrence of *Microcystis*. We are attempting to develop a biological control system to decrease the outbreak of *Microcystis* in a lake. With our system we can better understand the lifecycle of *Microcystis* (19, 24), which is probably regulated by several organic compounds derived from the cyanobacterium itself, and then develop a regulation method based on its lifecycle.

In previous studies, we found that *Microcystis* strains produced β-cyclocitral, 2-methyl-1-butanol and 3-methyl-1-butanol as VOCs (11, 18). It was determined for the first time that 2- and 3-methyl-1-butanols were excreted into the extracellular fraction of *Microcystis aeruginosa* NIES-102 (11, 18). The functions and biosynthesis of these alcohols in *M. aeruginosa* have not yet been elucidated. Watson (29) raised a concern as to whether the VOCs are signals or by-products. Furthermore, we observed this finding in the natural environment in 2008 and detected 2- and 3-methyl-1-butanols together with β-cyclocitral (unpublished result). According to our recent results using seven different genera of cyanobacteria, β-cyclocitral and the 2- and 3-methyl-1-butanols were produced only by *Microcystis* strains (12). In this study, we focused on the elucidation of production behavior and the biosynthetic mechanism of the 2- and 3-methyl-1-butanols.

While β-cyclocitral is biosynthetically produced from β-carotene, no origin was identified for the 2- and 3-methyl-1-butanols from the *Microcystis*. Branched-chain alcohols, including 3-methyl-1-butanol and 2-methyl-1-butanol, are known to be produced from the corresponding 2-keto acids by nitrogen starvation in yeast (26). The 2-keto acids are intermediates in the amino acid biosynthesis pathways and can be converted into aldehydes by broad-substrate-range 2-keto-acid decarboxylases (KDCs) and then to alcohols by alcohol dehydrogenases (ADHs) (1, 10, 21). 1-Propanol, 2- methyl-1-propanol, 1-butanol, and 2-phenylethanol are known to be generated from different 2-keto acids using the same steps (8). These branched-chain alcohols accumulate when the nutrients become limiting in *Saccharomyces* (9). It has been reported that 3-methyl-1-butanol induced the filamentation of *S. cerevisiae* with a global transcriptional response (2, 9, 13, 15).

In the present study, we attempted to elucidate the relationship among the growth of *Microcystis aeruginosa* NIES-843, nitrate nitrogen in a cultured broth and the production of the 2- and 3-methyl-1-butanols, and to clarify the enzymes involved in the synthesis of these alcohols in order to elucidate their function. Additionally, we report, for the first time, the genetic identification, purification and characterization of the *Microcystis* enzymatic activity responsible for the conversion of the 2-keto acids derived from amino acids into aldehydes.

*M. aeruginosa* NIES-843, whose genomic structure has been completed by Kaneko *et al.* (14), was obtained from the Microbial Culture Collection at the National Institute for Environmental Studies (NIES Collection, Ibaraki, Japan) and used in this study. The cyanobacterial strains were grown under 35 μmol m^−2^s^−1^ continuous illumination by fluorescent (cool white) light and shaking at 80 rpm in 400 mL CB medium at 30°C (23). To determine the growth curve of the cyanobacteria and the amount of nitrate nitrogen as the nitrogen source in the cultured broth, 10 mL cultured broth was collected. Three milliliters were then transferred to a quartz cell (1 cm) and the OD was measured at 660 nm using a V-530 UV/VIS spectrophotometer (JASCO, Tokyo, Japan). The remaining part was filtered using a GF/A filter (Whatman International, Maidstone, UK) and the nitrate nitrogen in the filtrate was measured using a Digital PACKTEST Multi (Kyoritsu Chemical Check Lab., Tokyo, Japan).

The collected cyanobacteria were directly analyzed as the total amount of VOC, and the filtrates were analyzed as the amount of released VOC in the medium. The intracellular amount of VOC was obtained by subtracting the VOC amount in the culture filtrate from the total VOC amount. A properly diluted aliquot (10 mL) of each sample was subjected to headspace solid-phase microextraction (HS-SPME) coupled with GC/MS for quantitative determination of the following volatile compounds: 2-methyl-1-butanol, 3-methyl-1-butanol, 2-phenylethanol, 1-propanol, 2-methyl-1-propanol, and 1-butanol (11).

*M. aeruginosa* NIES-843 was cultured well, and 10 mL each was collected at 0, 7, 14, 21, 28, 35, 42 d for VOC analysis. On day 14, the cell numbers of *M. aeruginosa* NIES-843 peaked in the stationary phase (Fig. 1a). As shown in Fig. 1b, nitrate nitrogen as the nitrogen source in the *Microcystis* culture was consumed by day 12. These VOCs began to increase around 21 d of culture (Fig. 1c, d and e), and the total maximum concentrations of the 2- and 3-methyl-1-butanols and 2-phenylethanol at 35 d were 47.3 ± 16.1, 107.7 ± 27.2 μg L^−1^, and 5.38 ± 2.56 mg L^−1^, respectively, and were also present in the culture filtrate. 1-Propanol was not detected, while 1-butanol and 2-methyl-1-propanol were detected at low concentrations (0.76 ± 0.03 and 9.72 ± 1.34 μg L^−1^, respectively). As a result, it was found for the first time that 2-phenylethanol was excreted into the extracellular fraction of *M. aeruginosa* NIES-843, which was produced in a higher concentration than the other branched-chain alcohols. Furthermore, the absolute configuration of 2-methyl-1-butanol released from the *Microcystis* was determined to be (S)-(-)-2-methyl-1-butanol using chiral GC/MS (Fig. S1), indicating that this is a single enantiomer derived from the corresponding L-isoleucine; therefore, 3-methyl-1-butanol and 2-phenylethanol were derived from L-leucine and L-phenylalanine, respectively, in the *Microcystis* (3).

No gene encoding a decarboxylase with activity toward branched-chain 2-keto acids in *Microcystis* has yet been cloned. Searching the entire genome of *M. aeruginosa* NIES-843 revealed three proteins (MAE36750, MAE50700, and MAE06010) with homology (34%, 24% and 22% identity, respectively) to the 2-keto-acid decarboxylase, KdcA, from *Lactococcus lactis* B1157 (25). Since MAE50700 and MAE06010 revealed high homology (77% and 83%, respectively) to IlvG and IlvB of *Synechocystis* sp. strain PCC6803, it is considered that these two enzymes are not 2-keto-acid decarboxylase. BLASTP homology search indicated that MAE36750 (called MaKDC) was homologous to the indolepyruvate decarboxylases and pyruvate decarboxylases found in various organisms. Highly conserved residues involved in catalysis and cofactor binding were observed in MaKDC, showing that MaKDC is a ThDP-dependent decarboxylase. *M. aeruginosa* NIES-843 genome DNA was obtained as described by Kaneko *et al.* (14). The MaKDC gene was amplified by PCR from the *M. aeruginosa* NIES-843 genome using a forward primer, 5′-TAGCCATGGCAATCACGATCGGCGAATA-3′ (*Nco*I site is underlined), and a reverse primer, 5′-TAGAGATCTAGCATGGGGTGAACGTAAAG-3′ (*Bgl*II site is underlined). The resultant PCR product was digested and cloned into the *Nco*I-*Bgl*II sites of pQE-60 (Qiagen, Hilden, Germany) to yield pQMDC1, in which MaKDC will be produced with the His_6_ tag at the C-terminus. The transformed *E. coli* M15 (Qiagen) was grown in 2×YT medium at 37°C in the presence of 75 μg L^−1^ ampicillin with shaking at 110 rpm. Production of the recombinant protein was induced by the addition of IPTG to the culture at an OD at 660 nm (OD_660_) of 0.6 to a final concentration of 2 mM, and the culture was allowed to grow for an additional 7 h at 30°C. Analyzing the VOC compounds in the culture of *E. coli* M15 expressing MaKDC, 2- and 3-methyl-1-butanol, and 2-phenylethanol were detected (Fig. 2) using the same method as above. The most abundantly detected compound was 2-phenylethanol, similar to that from the *Microcystis* culture.

The cells were harvested by centrifugation for 20 min at 4,000×*g* and then resuspended in sonication buffer (50 mM Tris [pH 8.0], 300 mM NaCl, 10 mM imidazole). After freezing and thawing, the cells were sonicated on ice (6 times for 10 s each) using a Tomy UD-201 sonicator and then centrifuged for 20 min at 20,000×*g*. The overexpressed protein was purified by nickel chelate chromatography (Qiagen). The sample was loaded onto the column and washed three times with washing buffer (50 mM Tris-HCl [pH 8.0], 300 mM NaCl, and 30 mM imidazole), and then the recombinant protein was eluted with the same buffer containing 250 mM imidazole. Fractions containing the protein were pooled, dialyzed against dialysis buffer (50 mM Tris [pH 8.0], 0.5 mM DTT, 0.1 mM EDTA, 50% glycerol), and then stored at −80°C. The size of the purified recombinant protein on the SDS-polyacrylamide gel was very consistent with the theoretical molecular mass of 61 kDa (Fig. 3).

To test the substrate specificity of the recombinant enzyme, the decarboxylation reactions of the 2-keto acids were performed. The decarboxylase activity of the purified enzyme was measured using 2,4-dinitrophenylhydrazine (DNPH, Panreac, Barcelona, Spain) as previously described (7, 16, 20). The substrates used to determine the specificity of the 2-keto-acid decarboxylase were 2-ketoisovalerate, 2-ketoisocaproate, 2-ketomethylvalerate, 2-ketomethyl-thiobutyrate, phenylpyruvate, indole-3-pyruvate and pyruvate. Typical 200 μL reactions containing 50 mM sodium phosphate buffer (pH 7.0), 30 mM substrate, 5 mM MgCl_2_, 1.5 mM thiamin diphosphate (ThDP) and MaKDC (10 μg L^−1^) and was incubated at 37°C for 20 min. For pyruvate and indole-3-pyruvate, 100 μg L^−1^ enzyme was added to the reaction mixture. Indole-3-pyruvate was preincubated at 25°C for 45 min, as recommended by Schutz *et al.* (22), and added to the reaction mixture at a 2 mM final concentration. All the samples were analyzed by reverse-phase high performance liquid chromatography (ELITE LaChrome System, Hitachi Hitech, Tokyo, Japan) using a 60% (v/v) acetonitrile/water mobile phase. Absorbance was measured at 365 nm. One enzyme activity unit was expressed as μmol of isobutyraldehyde produced per min per mg of protein.

As shown in Table 1, activity was the highest with 2-ketoisocaproate. The levels of decarboxylase activity for 2-ketomethylvalerate, 2-ketoisovalerate, and 2-phenylpyruvate when compared to 2-ketoisocaproate were 35.2, 74.3, and 64.8%, respectively. Decarboxylation activity with indole-3-pyruvate markedly decreased to 0.3%. No activity was detected with pyruvate even if 100-fold enzyme concentration was present in the reaction. The enzyme reactions were performed at different 2-ketoisovalerate concentrations (0.1, 0.5, 1, 1.5, 2, 2.5, 5, 7.5, 10 and 20 mM) under standard conditions. The *K_m_* and *k_cat_* values were calculated by fitting the data to the Lineweaver-Burk linear transformation of the Michaelis-Menten equation. The *K_m_*, *k_cat_*, and *k_cat_*/*K_m_* values for the enzyme were 0.52 mM, 7.22 s^−1^, and 13.88 s^−1^ mM^−1^, respectively.

In this paper, we reported the relationship among the growth of *M. aeruginosa* NIES-843, decrease of nitrate nitrogen in a cultured broth and production of VOCs, along with the genetic identification, purification, and characterization of a MaKDC in *M. aeruginosa* NIES-843. In a separate study, large amounts of these VOCs were detected together with the outbreak of cyanobacteria under natural conditions (unpublished results). Accordingly, it is considered that the 2- and 3-methyl-1-butanols and 2-phenylethanol are significant for *Microcystis*, because these alcohols are produced only from *Microcystis* using MaKDC, which shares homology with KDC involved in the lifecycle of yeast and are correlated with the death phase of the *Microcystis* lifecycle. In addition, this is the first report of 2-keto-acid decarboxylase producing the 2- and 3-methyl-1-butanols and 2-phenylethanol by an oxygenic photosynthetic microorganism. A future study should focus on the expressional regulation of the MaKDC gene under nutrition-limiting conditions and the 2- and 3-methyl-1-butanols and 2-phenylethanol as a function of the signaling molecule.

## Supplementary Material



## Figures and Tables

**Fig. 1:**
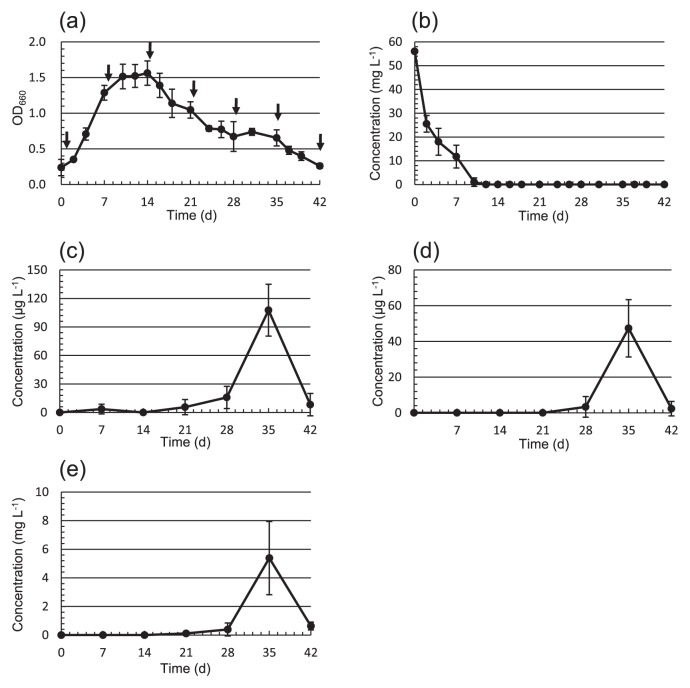
Temporal production of 3-methyl-1-butanol, 2-methyl-1-butanol, and 2-phenylethanol in *Microcystis aeruginosa* NIES-843. (a) Growth curve of *M. aeruginosa* NIES-843 measured at OD_660_. Arrow indicates the sampling time. Each value is the mean SD of triplicate measurements. (b) Nitrate nitrogen amounts are the mean SD of triplicate measurements. Quantitative determinations of (c) 3-methyl-1-butanol, (d) 2-methyl-1-butanol and (e) 2-phenylethanol from the culture filtrate of *M. aeruginosa* NIES-843 at each sampling time are the mean SD of triplicate measurements.

**Fig. 2:**
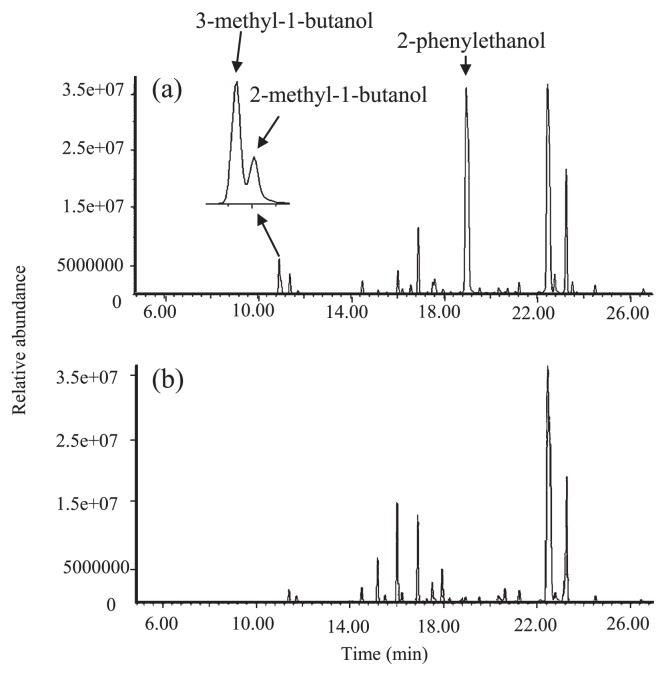
Detection of 2-methyl-1-butanol, 3-methyl-1-butanol and 2-phenylethanol from *Escherichia coli* M15 expressing 2-keto-acid decarboxylase (MaKDC). Comparison of the products between (a) *E. coli* M15 expressing MaKDC and (b) *E. coli* M15.

**Fig. 3:**
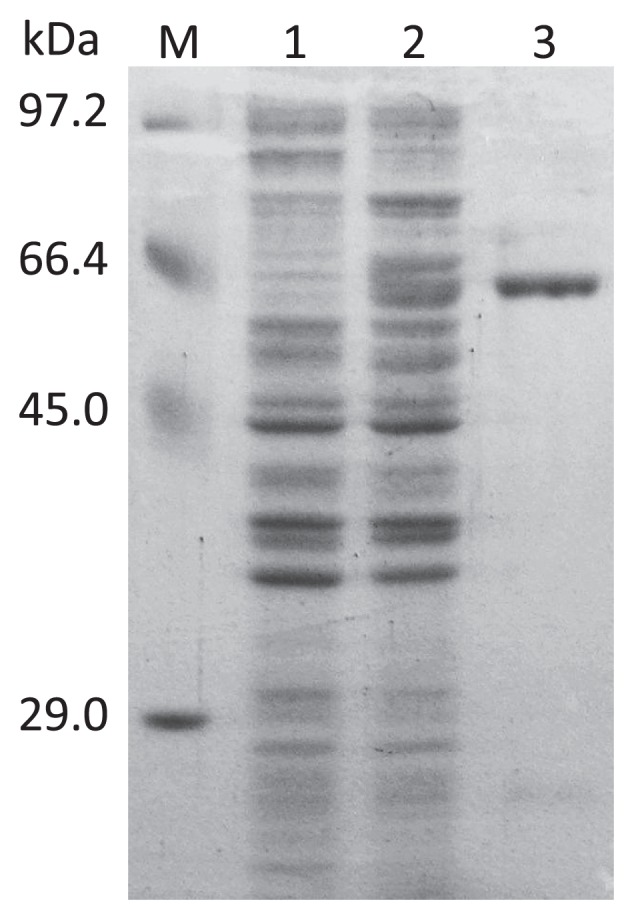
Coomassie brilliant blue (CBB)-stained SDS-polyacrylamide gel of purified 2-keto-acid decarboxylase (MaKDC). Lane 1, molecular mass marker. Lanes 2 and 3, total proteins of *E. coli* M15 harboring pQE-60 or pQMCD1 after induction, Lane 4, purified MaKDC obtained from the extract of lane 3.

**Table 1:** Substrate specificity of recombinant 2-keto-acid decarboxylase (MaKDC) activity

Substrate	Specific activity (U)	Relative activity (%)
2-ketoisocaproate	3.0 ± 0.1	100
2-ketomethylvalerate	1.0 ± 0.1	35.2
2-ketoisovalerate	2.2 ± 0.1	74.3
2-phenylpyruvate	1.9 ± 0.4	64.8
Pyruvate	Not detected	
Indole-3-pyruvate	0.009 ± 0.001	0.3
