# Supplementary material for: Volatile Organic Compounds Derived from 2-Keto-Acid Decarboxylase in Microcystis aeruginosa
Source: Microbes Environ. 2012 Oct 5;27(4):525–8. doi: 10.1264/jsme2.ME12099 (PMC4103566; doi:10.1264/jsme2.ME12099)
Supplement: Supplementary file 1 [file 27_525_s1.pdf]

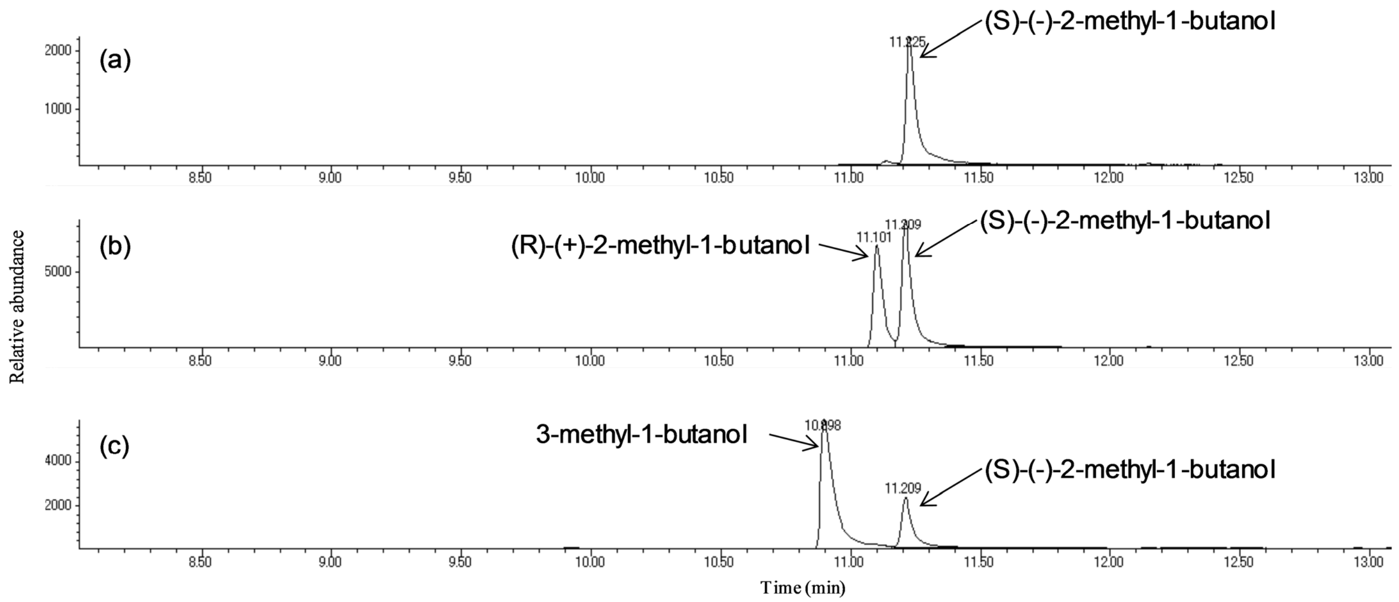


**Supplementary material**

**Fig. S1.** Selected ion monitoring (SIM) of *m/z* 70 of (a) commercially available (S)-(-)-2-methyl-1-butanol, (b) commercially available (±)-2-methyl-1-butanol and (c) 2-methyl-1-butanol and 3-methyl-1-butanol released from *Microcystis aeruginosa* NIES-843 by GC/MS using a chiral column, Beta DEX.
